# Gender difference in the impact of gynoid and android fat masses on the progression of hepatic steatosis in Japanese patients with type 2 diabetes

**DOI:** 10.1186/s40608-017-0163-3

**Published:** 2017-07-11

**Authors:** Ryotaro Bouchi, Tatsuya Fukuda, Takato Takeuchi, Yujiro Nakano, Masanori Murakami, Isao Minami, Hajime Izumiyama, Koshi Hashimoto, Takanobu Yoshimoto, Yoshihiro Ogawa

**Affiliations:** 1 Department of Molecular Endocrinology and Metabolism, Graduate School of Medical and Dental Sciences, Tokyo Medical and Dental University, 1-5-45 Yushima, Bunkyo-ku, Tokyo, 113-8519 Japan; 2 Center for Medical Welfare and Liaison Services, Tokyo Medical and Dental University, Tokyo, Japan; 3 Department of Preemptive Medicine and Metabolism, Graduate School of Medical and Dental Sciences, Tokyo Medical and Dental University, Tokyo, Japan; 4 CREST, Japan Agency for Medical Research and Development, Tokyo, Japan

**Keywords:** Gynoid, Android, Gender, Hepatic steatosis, Type 2 diabetes

## Abstract

**Background:**

Increased visceral adiposity is strongly associated with non-alcoholic fatty liver disease (NAFLD). However, little attention has been paid to the association between the change in subcutaneous adipose mass and the progression of non-alcoholic fatty liver disease (NAFLD). We aimed to investigate whether increased subcutaneous adipose tissue (gynoid fat mass) could be protective against the progression of NAFLD in Japanese patients with type 2 diabetes.

**Methods:**

This is a retrospective observational study of 294 Japanese patients with type 2 diabetes (65 ± 10 years old, 40% female). Liver attenuation index (LAI) measured by abdominal computed tomography was used for the assessment of hepatic steatosis. Both gynoid (kg) and android (kg) fat masses were measured by the whole body dual-energy X-ray absorptiometry. One-year changes in LAI, gynoid, and android fat masses were evaluated in both male and female patients. Linear regression analysis with a stepwise procedure was used for the statistical analyses to investigate the association of the changes in gynoid and android fat masses with the change in LAI.

**Results:**

LAI levels at baseline were 1.15 ± 0.31 and 1.10 ± 0.34 in female and male patients (*p* = 0.455). The change in gynoid fat mass was significantly and positively associated with the change in LAI in both univariate (standardized β 0.331, *p* = 0.049) and multivariate (standardized β 0.360, *p* = 0.016) models in the female patients. However, no significant association was observed in males. In contrast, the increase in android fat mass was significantly associated with the reduced LAI in both genders in the multivariate models (standardized β −0.651, *p* < 0.001 in females and standardized β −0.519, *p* = 0.042 in males).

**Conclusions:**

This study provides evidence that increased gynoid fat mass may be protective against the progression of NAFLD in female Japanese patients with type 2 diabetes.

## Background

Non-alcoholic fatty liver disease (NAFLD) has attracted attention for its association with cardio-metabolic risks [1, 2], atherosclerosis, and cardiovascular disease (CVD) [3–5] as well as with the progression of liver-specific diseases, including hepatic cirrhosis and hepatocellular carcinoma. Therefore, NAFLD has recently been recognized as a hepatic manifestation of metabolic syndrome [6, 7]. Diabetes is known to be a strong and independent risk factor for NAFLD [8]. Conversely, NAFLD has been histologically improved by the reduction of blood glucose level in patients with diabetes [9]. In addition, diabetes and insulin resistance are associated with histological severity of NAFLD in patients with normal transaminase levels [10]. These previous reports [8–10] suggest the importance of evaluating for NAFLD in diabetic patients, especially those with metabolic syndrome.

Regarding the body fat distribution, abdominal visceral fat has been reported to be more strongly associated with cardiovascular risks than body mass index (BMI), waist circumference, and abdominal subcutaneous fat [11, 12]. Also, it is important to evaluate body fat distribution, especially visceral adiposity in case of NAFLD because excess free fatty acids and chronic low-grade inflammation from visceral fat are considered to be two of the most important factors contributing to the progression of liver injury in NAFLD [13]. We have recently reported in a cross-sectional study that increased abdominal visceral fat is associated with hepatic fat accumulation regardless of BMI in Japanese patients with type 2 diabetes [14], suggesting that visceral adiposity may promote hepatic steatosis regardless of the weight of the person. Conversely, it has been reported that peripheral subcutaneous fat may have potential to be protective against accumulation of cardio-metabolic risks and ectopic fat [15, 16]. Peripheral subcutaneous fat may represent a “metabolic sink” for the storage of excess energy and may act against metabolic alterations and atherosclerosis [17, 18]. These studies suggest that accumulation of peripheral subcutaneous fat may act as a negative predictor for the progression of hepatic steatosis. However, it remains unclear so far whether increase in peripheral subcutaneous fat could be associated with the reduction of hepatic fat accumulation.

The whole body dual-energy X-ray absorptiometry (DXA) method provides more accurate measurements of the body composition and fat distribution than anthropometric parameters such as BMI [19]. A recent large scale epidemiological study from the United States [20] revealed that excess android fat mass (central obesity) was significantly associated with high triglycerides and low high-density lipoprotein (HDL) cholesterol levels in males and high low-density protein (LDL) cholesterol and low HDL cholesterol levels in females and excess gynoid fat mass (fat accumulation around the hips and bottom) showed a positive correlation with total cholesterol in males, whereas gynoid fat mass in females showed a favorable association with triglycerides and HDL cholesterol. These data suggest that the impact of android and gynoid fat masses on cardio-metabolic diseases including NAFLD may differ according to the gender. In this context, we sought to investigate the gender difference in the association of longitudinal changes in gynoid and android fat masses with the progression of NAFLD in Japanese patients with type 2 diabetes who are at a high risk for NAFLD.

## Methods

### Study design

This was a retrospective observational study to determine the impact of changes in regional fat mass (gynoid and android fat masses) with hepatic fat accumulation in Japanese patients with type 2 diabetes. All investigations were obtained from hospital records. The study protocol was in accordance with the principles of Declaration of Helsinki and was approved by the ethics committee of Tokyo Medical and Dental University.

### Subjects

Japanese patients with type 2 diabetes over 20 years of age who regularly visited to the outpatient clinic at Tokyo Medical and Dental University Hospital and had undergone the whole body DXA for the measurement of body fat distribution between July 1, 2012 and October 31, 2016 were enrolled in this study (*N* = 1524). Of the patients, we selected patients who had undergone the second measurement of DXA and abdominal computed tomography (CT) for the evaluation of hepatic steatosis twice with elapsed time of more than 9 months during the period (*N* = 342). We excluded patients with alcohol consumption ≥20 g/day in females and 30 g/day in males [21, 22], end-stage renal diseases (estimated glomerular filtration rate [eGFR] < 15 mL/min/1.73 m^2^), requiring renal replacement therapy, pregnancy, infectious diseases, and cancer. We also excluded patients who had received hepatotoxic drugs including glucocorticoids, tamoxifen, amiodarone, sodium valproate, and methotrexate, and those with other causes of liver diseases such as viral hepatitis [hepatitis B virus/ hepatitis C virus] and autoimmune liver diseases. The final sample included 294 patients in this retrospective study. The interval (median with interquartile range) between the first and second measurement of the DXA and abdominal CT were 1.02 (0.93–1.39) and 1.00 (0.90–1.09) years, respectively.

### Evaluation of gynoid and android fat masses

The total fat mass and non-fat mass, and the fat masses of android and gynoid regions were measured by the whole body DXA (Lunar iDXA, GE Healthcare, Madison, WI). Android and gynoid regions were defined as described in the past reports [23, 24]. The skeletal muscle mass index (SMI) was calculated by dividing skeletal muscle mass (fat-free mass in upper and lower extremities, kg) by height squared (m^2^). In this study, the existing reports of the patients where the DXA has been done during 2012 and 2016 were used.

### Evaluation of hepatic fat accumulation

Hepatic fat accumulation was determined by LAI in the abdominal CT examination (Aquilion PRIME, Toshiba Medical Systems, Tochigi, Japan) as described previously [24, 25]. Briefly, both hepatic and splenic attenuation values were measured on non-contrast CT scans by using eight circular ROI cursors with a diameter of 1.5 cm in the liver and 3 in the spleen. In the liver, four ROIs were located in each of the right anterior, right posterior, left medial, and left lateral segments. Then, the average attenuation value of liver (eight points) divided by average attenuation value of spleen (three points) was defined as LAI in this study. We assessed the visceral fat area (VFA) and subcutaneous fat area (SFA) using CT as previously reported [14].

### Clinical and biochemical analysis

Information on alcohol intake, smoking, medication and past history were obtained from medical records. Smoking history was categorized as non-smoker or current smoker. Information about the previous CVD and diabetic retinopathy were obtained based on the medical records. The latex agglutination method was used for the measurement of HbA1c. HbA1c values estimated as the Japan Diabetes Society (JDS) method was converted to the National Glycohemoglobin Standardization Program values [26]. BMI was calculated as weight in kilograms divided by height in meters squared (kg/m^2^). Systolic and diastolic blood pressures (SBP and DBP) were measured after 5 min of seated rest, using an electronic sphyngomanometer (ES-H55, Terumo Inc., Tokyo, Japan). Grip strength (kg) was measured using a hand dynamometer Grip-D (TKK5401, Takei, Niigata, Japan). We defined muscle strength as average of grip strength in this study. Urinary albumin and creatinine concentrations were measured using turbidimetric immunoassay and enzymatic method, and the ratio of urinary albumin-to-creatinine ratio (ACR, mg/g) was calculated for the assessment of albuminuria in a spot urine sample. GFR (ml/min/1.73 m^2^) was calculated using the equation for the Japanese [27].

### Statistical analysis

Statistical Analysis was carried out using SPSS software (version 21.0; IBM Corp. Released 2012. IBM SPSS Statistics for Windows, Armonk, NY: IBM Corp.), and the results were expressed as mean ± standard deviation (SD), median and interquartile range (IQR) or percentages. Differences between male and female patients were tested with a t-test or Mann-Whitney U test for continuous variables or chi-square test for categorical variables, as appropriate. Normality was tested by the Kolmogorov-Smirnov test. Linear regression analysis in a stepwise manner was carried out to identify the longitudinal association of changes in gynoid and android fat masses with that in LAI with a duration of one year. Putative risk factors examined were duration of diabetes, BMI, HbA1c, triglycerides/high-density lipoprotein cholesterol (TG/HDL-C) ratio, alanine aminotransferase (ALT), eGFR, urinary ACR and the use of insulin, oral hypoglycemic agents, angiotensin-receptor blockers, and statins. Age was forced into the model because aging is associated with alterations in the amount and distribution of body fat depots with a shift from subcutaneous to visceral fat accumulation [28] and increases the risk for the progression of ectopic fat accumulation including fatty liver [29]. All *p* values less than 0.05 were considered statistically significant.

## Results

### Clinical characteristics

A total of 294 Japanese patients with type 2 diabetes (mean age 65 ± 10 years; 40% female) were enrolled in this study. Table 1 shows the clinical characteristics by gender. Female patients had significantly lower SBP and DBP, higher HDL-C and low-density lipoprotein cholesterol (LDL-C) and lower uric acid and gamma-glutamyl transpeptidase (γ-GTP) levels than male patients. There were no significant differences in glycemic control (HbA1c levels), duration of diabetes and prevalence of diabetic microvascular complications (retinopathy and nephropathy) between the two groups. Regarding the body composition, female patients had a significantly lower grip strength, SMI, total non-fat mass, VFA, and higher percent body fat and SFA levels than male patients. LAI levels were comparable between females and males. The prevalence of previous CVD was significantly lower in females than that in males and females were less likely to receive anti-platelet agents. No significant differences were observed in the prescription rate of diabetic medications by gender.

**Table 1:** Baseline characteristics by gender

	Female (*N* = 116)	Male (*N* = 178)	*P* values
Age (years)	65 ± 9	65 ± 10	0.915
SBP (mmHg)	125 ± 15	129 ± 14	0.032
DBP (mmHg)	72 ± 14	77 ± 12	0.002
Body mass index (kg/m^2^)	24.5 ± 4.7	25.0 ± 4.2	0.314
Grip strength (kg)	18.7 ± 5.4	31.5 ± 8.2	< 0.001
Skeletal muscle index	5.9 ± 0.9	7.2 ± 1.1	< 0.001
Total fat mass (kg)	22.2 ± 8.6	21.1 ± 8.2	0.281
Total non-fat mass (kg)	33.8 ± 5.4	46.5 ± 7.1	< 0.001
Andoroid (kg)	2.0 ± 1.0	2.2 ± 1.1	0.104
Gynoid (kg)	3.3 ± 1.2	2.9 ± 1.3	0.004
Andoroid (%)	8.3 ± 1.9	9.8 ± 1.8	< 0.001
Gynoid (%)	15.0 ± 2.8	13.4 ± 1.4	< 0.001
Body fat (%)	38.8 ± 7.4	30.3 ± 6.8	< 0.001
VFA (cm^2^)	119 ± 61	150 ± 71	< 0.001
SFA (cm^2^)	192 ± 88	145 ± 86	< 0.001
Liver attenuation index	1.15 ± 0.31	1.10 ± 0.34	0.445
Duration of diabetes (years)	6 (5–7)	8 (6–9)	0.059
HbA1c (mmol/mol)	54 ± 12	57 ± 11	0.143
HbA1c (%)	6.9 ± 1.5	7.2 ± 1.4	0.143
Triglycerides (mmol/l)	1.58 (1.7–1.80)	1.74 (1.56–1.80)	0.275
HDL cholesterol (mmol/l)	1.65 ± 0.55	1.45 ± 0.40	< 0.001
LDL cholesterol (mmol/l)	3.16 ± 0.89	2.77 ± 0.72	0.012
AST (U/l)	29 (25–33)	28 (23–30)	0.605
ALT (U/l)	28 (23–32)	29 (26–33)	0.556
γ-GTP (U/l)	46 (36–57)	64 (54–74)	0.020
Uric acid (μmol/l)	284 (271–297)	337 (325–348)	< 0.001
eGFR (ml/min/1.73 m^2^)	74.8 ± 23.3	72.9 ± 20.8	0.456
Log ACR (mg/g)	1.59 ± 0.59	1.52 ± 0.62	0.355
PDR (%)	8	5	0.217
History of CVD (%)	5	15	0012
C-reactive protein (mg/l)	0.19 (0.16–0.25)	0.21 (0.13–0.28)	0.716
Insulin (%)	26	24	0.801
Sulfonylureas (%)	11	16	0.263
Biguanides (%)	24	23	0.859
Alpha-GIs (%)	3	7	0.218
Glinides (%)	4	3	0.689
TZDs (%)	3	4	0.820
DPP4 inhibitors (%)	29	30	0.815
GLP1 receptor agonists (%)	1	2	0.366
SGLT2 inhibitors (%)	0	1	0.851
ARBs (%)	32	38	0.290
Calcium channel blockers (%)	32	34	0.750
Statins (%)	37	31	0.347
Anti-platelet agents (%)	8	17	0.023

*Abbreviations*: *ACR* albumin-to-creatinine ratio, *ALT* alanine transaminase, *ARBs* angiotensin receptor blockers, *AST* aspartate transaminase, *CVD* cardioivascular disease, *DBP* diastolic blood pressure, *DPP4* dipeptidyl peptidase 4, *eGFR* estimated glomerular filtration ratio, *GIs* glycosidase inhibitors, *GLP1* glucagon-like peptide-1; GTP, glutamyl transpeptidase, *HDL* high-density lipoprotein, *LDL* low-density lipoprotein, *PDR* proliferative diabetic retinopathy, *SBP* systolic blood pressure, *SFA* subcutaneous fat area, *SGLT2* sodium-glucose cotransporter 2, *TZDs* thiazolidinediones, *VFA* visceral fat area

### Association of changes in gynoid and android with progression of hepatic steatosis

As shown in Table 2, change in gynoid fat mass was significantly and positively associated with LAI in female but not male patients with type 2 diabetes in univariate linear regression models. After adjusting for covariates including TG/HDL-C ratio, statistical significance between gynoid fat mass and LAI remained unchanged in female patients. In contrast, gynoid fat mass was not associated with the change in LAI in male patients in the multivariate model. As expected, the change in android fat mass was significantly associated with the change of LAI in both gender in the univariate models. In the multivariate models, increase in android fat mass was significantly associated with the progression of hepatic steatosis in both genders.

**Table 2:** Linear regression analysis of liver attenuation index according to changes in gynoid and android stratified by gender

	Female (*N* = 116)		Male (*N* = 178)	
	Standardized β	*P* values	Standardized β	*P* values
Univariate	(Adjusted R^2^ = 0.15)		(Adjusted R^2^ = 0.00)	
ΔGynoid (%)	0.421	0.003	0.135	0.359
Multivariate model	(Adjusted R^2^ = 0.40)		(Adjusted R^2^ = 0.24)	
ΔGynoid (%)	0.473	0.003	0.157	0.249
eGFR	0.474	0.003	NA	
ΔHbA1c	−0.323	0.032	−0.274	0.045
TG/HDL-C ratio	NA		−0.309	0.031
ΔTG/HDL-C ratio	NA		−0.263	0.056
ARBs	NA		0.240	0.077
Univariate	(Adjusted R^2^ = 0.24)		(Adjusted R^2^ = 0.13)	
ΔAndroid (%)	−0.515	< 0.001	−0.385	0.007
Multivariate model	(Adjusted R^2^ = 0.50)		(Adjusted R^2^ = 0.23)	
ΔAndroid (%)	−0.548	< 0.001	−0.299	0.018
TG/HDL-C ratio	−0.446	0.002	−0.388	0.016
Biguanides	0.403	0.005	NA	
ΔTG/HDL-C ratio	NA		−0.270	0.048

*Abbreviations*: *ARBs* angiotensin receptor blockers, *eGFR* estimated glomerular filtration ratio, *HDL-C* high-density lipoprotein cholesterol, *TG* triglycerides

### Gender difference regarding the correlation of changes in gynoid and android fat masses with changes in markers for body composition and cardio-metabolic risks

Table 3 shows the correlation of changes in gynoid and android fat masses with changes in markers for body composition (LAI, VFA, and SFA) and cardio-metabolic risks by gender. Increased android fat mass was significantly correlated with hepatic fat (LAI), abdominal visceral (VFA) and subcutaneous (SFA) fat accumulation in both gender. The positive association between gynoid fat mass and LAI was significant in females but not in males. In female, android fat mass was positively and gynoid fat mass was negatively correlated with change in HbA1c, AST, ALT and gamma-GTP. Increased HDL-C was inversely associated with change in android fat mass and positively associated with change in gynoid fat mass in females. On the other hand, increased android fat mass was negatively correlated with HDL-C and positively correlated with TG/HDL-C ratio in male patients. No significant association was observed between gynoid fat mass and HbA1c, TG, HDL-C, AST, and ALT in males.

**Table 3:** Correlation of changes in android and gynoid with changes in markers for body composition and cardio-metabolic risks according to gender

	Female				Male			
	⊿Android (%)		⊿Gynoid (%)		⊿Android (%)		⊿Gynoid (%)	
	r	*P* values	r	*P* values	r	*P* values	r	*P* values
⊿LAI	−0.247	0.050	0.340	0.006	−0.292	0.042	0.131	0.221
⊿VFA (cm^2^)	0.595	< 0.001	−0.218	0.086	0.429	< 0.001	−0.185	0.080
⊿SFA (cm^2^)	0.329	0.008	−0.137	0.285	0.422	< 0.001	−0.136	0.198
⊿HbA1c (%)	0.345	0.005	−0.395	0.001	−0.018	0.849	−0.069	0.461
⊿TG (mmol/l)	0.108	0.388	0.056	0.658	0.172	0.067	−0.070	0.455
⊿HDL-C (mmol/l)	−0.354	0.004	0.141	0.260	−0.303	0.001	−0.055	0.562
⊿CRP (mg/l)	0.129	0.301	−0.101	0.421	0.065	0.493	−0.022	0.812
⊿UA (μmol/l)	0.040	0.751	0.089	0.476	0.151	0.108	0.168	0.172
⊿AST (U/l)	0.305	0.013	−0.243	0.049	0.037	0.692	−0.130	0.165
⊿ALT (U/l)	0.372	0.002	−0.333	0.006	0.102	0.279	−0.071	0.451
⊿γ-GTP (U/l)	0.334	0.006	−0.336	0.006	0.232	0.013	−0.175	0.041
⊿TG/HDL-C ratio	0.193	0.120	0.040	0.752	0.193	0.039	−0.013	0.892
⊿BMI (kg/m^2^)	0.294	0.015	−0.095	0.440	0.556	< 0.001	−0.159	0.088

*Abbreviations*: *ALT* alanine transaminase, *AST* aspartate transaminase, *BMI* body mass index, *CRP* C-reactive protein, *γ-GTP* gamma-glutamyl transpeptidase, *HDL-C* high-density lipoprotein cholesterol, *LAI* liver attenuation index, *SFA* subcutaneous fat area, *TG* triglycerides, *UA* uric acid, *VFA* visceral fat area

## Discussion

We demonstrated for the first time that increase in gynoid fat mass is positively and increase in android fat mass is negatively associated with change in LAI measured by abdominal CT in female patients with type 2 diabetes. In contrast, no significant longitudinal association was observed between gynoid fat mass and LAI in male patients. Regional fat accumulation has recently attracted attention for its differential impact on the cardio-metabolic risk and atherosclerosis. Okauchi et al. previously reported that reduction of visceral fat is associated with decrease in the number of components of metabolic syndrome in Japanese men [30]. NAFLD is thought to be a hepatic manifestation of metabolic syndrome [6, 7]. Therefore, it is conceivable that management of visceral adiposity is important to reduce the risk for metabolic syndrome, NAFLD, and future cardiovascular events. However, little has been known so for whether change in subcutaneous fat (gynoid fat mass) could be associated with the progression of hepatic steatosis. We have previously reported in a cross-sectional study that android-to-gynoid fat mass (A/G) ratio is strongly correlated with VFA and insulin resistance in patients with type 2 diabetes, and A/G ratio is significantly associated with the prevalent NAFLD and increased risk for carotid atherosclerosis [24]. In this study, we revealed the gender difference in longitudinal association of gynoid and android fat masses with the progression of NAFLD in patients with type 2 diabetes who are at a high risk for the initiation and progression of NAFLD.

Although it is unclear why gynoid fat mass is shown to be protective against the progression of NAFLD only in female patients in this study, estrogen levels may affect body fat distribution and hepatic fat accumulation in females. Estrogen receptors α knockout mice have increased amounts of visceral adipose tissue and hepatic fat accumulation [31]. These mice also shows adipocyte hyperplasia and hypertrophy, insulin resistance, and glucose intolerance [32]. It has been reported that NAFLD is more prevalent in post-menopausal women than pre-menopausal women and worsens after menopause [33]. Considering these previous studies [31–33], energy excess in premenopausal female may be likely to flow into the subcutaneous fat depot (increase in gynoid fat mass); accordingly, hepatic fat accumulation could be repressed. We also revealed that increase in gynoid fat mass was significantly correlated with the reduction of HbA1c, AST, ALT, and gamma-GTP in females (Table3). These findings imply that hepatic inflammation in the liver can improve in parallel with the increase in gynoid fat mass, presumably leading to improvement of glycemic control in females. In contrast, we found no gender difference in the significant association between android and the progression of NAFLD (Tables 2 and 3). It makes a great deal of sense because visceral fat accumulation promotes the production of free fatty acid, inducing hepatic de novo lipogenesis and abnormal secretion of adipokines including adiponectin, leptin, and interleukin-6 [34] which can exacerbate both chronic inflammation and insulin resistance [35].

Other than changes in body fat composition, we found worsening of glycemic control per se was independently associated with the progression of NAFLD in both genders (Table 2). The finding is consistent with previous studies [8–10]. Diabetes accelerates the pathology of nonalcoholic steatohepatitis in the type 2 diabetic rat model [36] and human [37]. It is therefore important to achieve good glycemic control in patients with diabetes for the prevention and improvement of NAFLD. We further found that not only baseline level of TG/HDL-C ratio, a surrogate marker for insulin resistance [38], but also change in TG/HDL-C ratio were significantly associated with the change in hepatic steatosis. Insulin resistance has been reported to be associated with NAFLD regardless of glucose levels [39] and patients with NAFLD have reduced insulin sensitivity in the muscle, liver, and adipose tissue. It is clearly revealed that increased insulin resistance promotes the hepatic lipid synthesis, resulting in the initiation and progression of NAFLD [40].

We would like to emphasize the fact that the whole body DXA can be used for the simultaneous assessment of both android (abdominal fat) and gynoid (fat accumulation around the hips and bottom) fat masses with low cost and low risk for exposure to radiation compared to CT. In this study, change in android fat mass was significantly correlated with changes in VFA and SFA in both genders (Table 3). In contrast, gynoid fat mass was not associated with VFA or SFA. These findings suggest that android fat mass is reliable for evaluating visceral adiposity and gynoid fat mass is distinct from android fat mass, VFA, and SFA and measurement of gynoid fat mass can aid us in further understanding the relationship between body fat distribution and the obesity-related conditions such as metabolic syndrome, NAFLD and CVD.

### Limitations

Our study has several limitations. First, this is a hospital-based study and consist of only Japanese patients with type 2 diabetes; thus generalizability of the results is limited. Second, histological findings are not available. Third, we used indirect methods (LAI) to estimate hepatic fat. It is therefore to be determined in future studies whether change in gynoid fat mass could be associated with triglycerides contents measured by magnetic resonance spectroscopy. In addition, it is to be elucidated whether change in gynoid fat mass could be associated with hepatic inflammation or fibrosis in the future studies. Fourth, follow-up period of the DXA is relatively short. Finally information on diet and exercise both of which could affect body fat composition and hepatic fat accumulation is not available in this study.

## Conclusions

In conclusion, our data suggest that female Japanese patients with type 2 diabetes with increased gynoid fat mass are at a low risk for the progression of NAFLD and increase in android fat mass is positively associated with hepatic fat accumulation in both genders.

## Abbreviations

ACR: Albumin-to-creatinine ratio
ALT: Alanine aminotransferase
AST: Aspartate aminotransferase
BMI: Body mass index
CT: Computed tomography
CVD: Cardiovascular disease
DBP: Diastolic blood pressure
DXA: Dual-energy X-ray absorptiometry
eGFR: Estimated glomerular filtration rate
HDL: High-density lipoprotein
IQR: Interquartile range
JDS: Japan Diabetes Society
LAI: Liver attenuation index
LDL: Low-density protein
NAFLD: Non-alcoholic fatty liver disease
ROI: Regions of interest
SBP: Systolic blood pressure
SD: Standard deviation
SFA: Subcutaneous fat area
SMI: Skeletal muscle index
TG: Triglycerides
VFA: Visceral fat area
γ-GTP: Gamma-glutamyl transpeptidase
